# Heart Failure: Is There an Ideal Biomarker?

**DOI:** 10.31083/j.rcm2411310

**Published:** 2023-11-09

**Authors:** Giuseppe Clemente, John Sebastian Soldano, Antonino Tuttolomondo

**Affiliations:** ^1^Internal Medicine and Stroke Care Ward, University Hospital Policlinico P. Giaccone, 90127 Palermo, Italy

**Keywords:** heart failure, pathophysiology, biomarkers, natriuretic peptides, hs-cTnT

## Abstract

An always-rising prevalence of heart failure (HF), formerly classified as an emerging epidemic in 1997 and still representing a serious problem of public health, imposes on us to examine more in-depth the pathophysiological mechanisms it is based on. Over the last few years, several biomarkers have been chosen and used in the management of patients affected by HF. The research about biomarkers has broadened our knowledge by identifying some underlying pathophysiological mechanisms occurring in patients with both acute and chronic HF. This review aims to provide an overview of the role of biomarkers previously identified as responsible for the pathophysiological mechanisms subtending the disease and other emerging ones to conduct the treatment and identify possible prognostic implications that may allow the optimization of the therapy and/or influence a closer follow-up. Taking the high prevalence of HF-associated comorbidities into account, an integrated approach using various biomarkers has shown promising results in predicting mortality, a preferable risk stratification, and the decrease of rehospitalizations, reducing health care costs as well.

## 1. Introduction

Although the prevalence and incidence of heart failure (HF) differ in different countries, depending on differences in study designs, it remains a disease with high prevalence and mortality [1]. The real prevalence of the disease is underestimated and increases with age [2]. Moreover, considering the general demographic data, it can be considered increasing in all Western countries where it continues to represent the leading cause of hospitalization and, despite the treatments we have and the apparent reduction in the number of hospital admissions, the rehospitalization rate remains high [3]. In fact, in developed countries, the age-corrected incidence may seem to be decreasing, an expression of increasingly effective management but the aging of the population accounts for its overall increase [4]. As derived from the data of the Italian National Outcomes Program, prior to the introduction of SGLT2 and 1 inhibitors (sodium-glucose- cotransporter 2 and 1 inhibitors), no therapeutic agent had been demonstrated in recent years to significantly reduce one-month rehospitalization rates and 1- and 5-year mortality rates. Considering the pathophysiological assumptions and results achieved by recent studies, it is reasonable to think of a further improvement in the prospects of care and the performance status of these patients [5, 6]. A better knowledge of the alterations of the neuro-hormonal and cytokine balance underlying its genesis and maintenance could contribute significantly to this. It is, therefore, desirable to identify biomarkers that can allow a practical clinical approach to identify subjects most at risk deserving of closer follow-up and possible optimization of therapies. Intravenous iron infusion also reduced the composite risk of first hospitalization and recurrent hospitalizations for HF, although with no effect on mortality [7]. Therefore, even the biomarkers of the iron metabolism could represent, indirectly, an index of evaluation of the state of the patient suffering from HF. It could also be useful to understand the role of individual biomarkers in the different categories of HF (heart failure with mildly reduced ejection fraction (HFmrEF), heart failure with preserved ejection fraction (HFpEF), heart failure with reduced ejection fraction (HFrEF)) and to investigate the biohumoral modifications that will follow the achievement of new metabolic and neurohumoral balances with the use of new drugs. Cardiac markers analyzed in this review range from having both diagnostic and prognostic capabilities but are increasingly being studied as therapeutic targets in order to impact the evolution of the disease itself.

## 2. Definition

European Society of Cardiology (ESC) Guidelines 2021 trace HF back to its clinical expression of symptoms (breathlessness, ankle swelling, fatigue) and signs (elevated jugular venous pressure, pulmonary crackles, peripheral edema) resulting from reduced cardiac output and/or high filling pressures caused by a structural or functional cardiac abnormality [4]. Braunwald, on the other hand, offers a definition that underlies a pathophysiological framework and anticipates the conditions for compensatory responses to the disease that result, among other things, in the production of biomarkers that are an expression of pathophysiological adaptation. In addition to the usefulness in understanding pathogenetic mechanisms, biomarkers can support therapeutic choices and offer prognostic implications if considered in combination [8]. In 1999 the full definition of biomarkers was clarified during the consensus conference of the Food and Drug Administration (FDA)/National Institutes of Health on “Biomarkers and Surrogate Endpoints: Advancing Clinical Research and Applications which qualifies them as a characteristic that is objectively measured and evaluated as an indicator of normal biologic processes, pathogenic process, or pharmacologic responses to a therapeutic intervention” [9]. The appropriate application of biomarkers could translate into substantial benefits, making them fundamental for the rational development of therapeutic strategies [10].

## 3. Pathophysiology

Regardless of the specific etiology of HF, the disease is unified by shared pathophysiological responses, which involve compensatory mechanisms that, over time, may ultimately impair cardiac function, culminating in clinical manifestations once these compensatory mechanisms are exhausted. Specifically, any condition leading to a structural or functional alteration of the myocardial tissue can induce HF. In turn, the body responds with the activation of several adaptative mechanisms involving the sympathetic nervous system, biohumoral responses, cytokine release, and hydro-electrolytes balance adjustments. However, sustained and prolonged stress on these systems can eventually lead to a state of overt decompensation, initially under stress and later even at rest [11]. As a result of these alterations, peripheral hypoperfusion occurs. When the amount of oxygenated blood is not sufficient to meet the metabolic needs of the cells, there is first an increased extraction of oxygen from the arterial blood that perfuses the tissues. Subsequently, a redistribution of cardiac output is carried out involving the aforementioned systems. Decreased efficiency of cardiac contraction and redistribution of volume lead to venous congestion. These conditions result in a very complex syndrome that makes it imperative to know the underlying pathophysiological mechanisms in order to be able to carry out the necessary therapeutic interventions to maintain in a range as physiological as possible the adaptations of the response to myocardial dysfunction [12]. The expression of tissue dysfunction and adaptation mechanisms translates into the production and release into circulation of substances that represent HF, not only markers but also therapeutic targets and, above all, clinical monitoring and risk stratification [13]. In this context, it is mandatory to maintain a holistic view of the disease in order to intervene in different phases of its natural history and maintain the adaptation of the organism as physiological as possible [14], and for this purpose, biomarkers can represent an important point of reference.

## 4. Biomarkers: Historical Reviews, State of Art and New Frontiers

As already mentioned, some biomarkers can be considered as the expression of the body’s adaptation and response mechanisms to HF, mechanisms and responses that, in part, are common to all forms of circulatory failure. But the history of cardiac biomarkers begins in 1954 with the identification of myocardial necrosis biomarkers: first, aspartate aminotransferase (AST) was identified, and then lactate dehydrogenase (LDH) and creatine kinase (CK), all of which were not very specific. With the identification of cardiospecific CK and LDH isoenzymes, the road to specificity was traced. In 1979, the World Health Organization (WHO) introduced, among the criteria for the diagnosis of acute myocardial infarction (AMI), the serial elevation of serum concentrations of cardiac enzymes [15]. In 1990, data on the diagnostic capacities of cardiac troponin T (cTnT) and cardiac troponin I (cTnI), structural components of the thin filaments of the cardiac striated muscle, and cardio specific isoforms in AMI were reported. The natriuretic peptides (NPs) released by cardiomyocytes have been widely demonstrated as markers for the diagnosis and risk stratification in HF [13]. Although historically, troponin and NPs are considered the reference markers of acute coronary syndrome and, respectively, HF [16], their combined assessment can offer a better diagnostic, prognostic, and monitoring contribution by identifying patients at higher risk [17]. However, new data are emerging that question the role of NPs as a guide in the management of HF [18], while the high-sensitivity troponins and soluble suppression of tumorigenesis-2 would seem to represent more reliable biomarkers for risk stratification. This is explained further in the specific sections and particularly concerns HFpEF. Also, in heart valve disease, biomarkers could play a role in the stratification of patients’ risk and help identify the optimal time of cardiac surgery by identifying the early stages of HF and avoiding further structural alteration [19]. Other biomarkers evaluated as predictors of adverse outcomes are galectin-3, growth differentiation factor 15, mid-regional pro-adrenomedullin, and markers of renal dysfunction. Genomic, transcriptomic, proteomic, and metabolomic investigations could further improve the overall approach to HF [13].

### 4.1 The Natriuretic Peptide System

NPs are biologically active molecules, secreted in the forms of Atrial Natriuretic Peptide (ANP) and Brain Natriuretic Peptide (BNP) by the heart, long used in the evaluation of the degree of congestion in HF. ANP, BNP, and CNP (C-type natriuretic peptide, initially identified in the brain and whose levels do not change much with cardiac overload, probably has a role in the regulation of vascular tone with paracrine action) [20] are degraded by endopeptidase neprilysin [21]. While both N-terminal prohormone of BNP (NT-proBNP) and BNP are derived from ProBNP degradation, only BNP is further degraded by neprilysin. As a result, the neprilysin inhibitor sacubitril directly affects only BNP levels, that transiently increase when treatment is initiated, while NT-proBNP levels tend to reduce over time, likely due to the indirect hemodynamic effect of the treatment [22]. They can, in all respects, be considered cardiac hormones sanctioning, therefore, the endocrine function of the heart. The receptor with the greatest affinity seems to be the natriuretic peptide receptor-A (NPR-A or guanylyl cyclase-A), whose link with ANP and BNP increases intracellular cyclic guanosine monophosphate (cGMP) with similar effects on target cells [23]. Other receptors identified are NPR-B or guanylyl cyclase-B and the NPR-C or clearance receptor. NPR-C removes natriuretic peptides from circulation. The generation of intracellular cGMP resulting from the binding of NPs with NPR-A and NPR-B determines their interaction with specific enzymes and ion channels [24]. ANP is secreted from the cardiac atria, and BNP is secreted from the cardiac ventricles in response to increased diastolic wall stress. Both ANP and BNP contribute to natriuresis and vasodilation through endocrine and paracrine mechanisms, resulting in a decrease in aldosterone secretion, reduction in renal-tubular sodium reabsorption, lowered blood pressure and mitigation of cardiac hypertrophy and ventricular fibrosis [25, 26]. These are, therefore, peculiar analytes as they have protective effects, unlike the molecules that are usually dosed and that are an expression of organ damage (e.g., troponin). ANP has a shorter half-life than BNP and NT-proBNP, making the latter more advantageous in clinical practice in diagnostic and prognostic terms [24]. In some patients, serum levels of NP can be low due to several factors now established as obesity, polymorphisms in the natriuretic peptide B (*NPPB*) gene, African ancestry, hypercortisolism, increased androgenicity in women, insulin resistance, and certain medications [27]. In addition, also normal values of NT-pro-BNP in patients with HFpEF are more associated with adverse events, including increased mortality [28]. Furthermore, although SGLT2 inhibitors have no significant effect on NPs levels, they reduce hospital admissions and improve the performance status of patients with HFpEF. For these reasons, BNP may not serve as an ideal biomarker for HFpEF management [29]. In addition, while serum NT-pro-BNP levels correlate with left ventricle (LV) end-diastolic wall stress and elevations in the pulmonary capillary wedge pressure (PCWP), some patients with HFpEF do not exhibit significant increases in BNP levels despite high terminal diastolic pressure in the LV [30, 31]. However, given that dyspnea is the primary symptom of left ventricular failure, BNP and NT-pro-BNP are often employed in the differential diagnosis between cardiac and respiratory diseases [32]. The data of the *Breathing Not Properly Multinational Study* showed that at levels below 50 pg/mL, BNP had a negative predictive value of 96%, representing, therefore, a good test for rule out in the acute setting [33]. The TIME-CHF trial (Trial of Intensified (BNP-guided) versus standard (symptom-guided)) showed that patients receiving BNP-guided therapy had a lower rate of hospitalizations compared to those receiving symptom-guided standard therapy. However, the trial did not demonstrate a significant improvement in hospitalization-free survival time or quality of life [34, 35]. In addition, we must consider the increases in NPs in patients with kidney disease and the worsening of renal function in patients with HF, which opens a great chapter of cardio-renal syndromes and which suggests the need for new findings to establish their diagnostic and prognostic validity [36]. Other factors may cause changes in the levels of NPs in addition to kidney disease, neprilysin inhibitors (in the latter case except, as already mentioned, for NT-proBNP) — (Table 1). Increases in NT-proBNP could also identify patients most at risk of sudden cardiac deaths in the preclinical phase of HF, as demonstrated by prospective design studies [37]. Another promising molecule could be Corin, which represents a pro-natriuretic peptide convertase. This molecule, even in its enzymatically inactive form, has been shown to have protective effects on the myocardium. Therefore, the evaluation of serum Corin levels could represent a biomarker of disease progression that precedes the alterations of the other NPs together with which it regulates fluid homeostasis in HF [38]. This could determine an earlier therapeutic intervention preventing disease progression and clinical expression, especially if low Corin concentrations are combined with impaired pro-ANP cleavage and, subsequently, very high levels of NPs [39]. However, further studies are needed to better understand the role of Corin in fluid homeostasis and the reduction of its serum levels in relation to augmented plasma levels of NPs due to enzymatic downregulation [40, 41]. The function of Corin is strongly correlated with that of the ANP. In fact, Corin actives the ANP precursor to mature ANP. Activated ANP deficiency causes heart disease and hypertension [42]. We foresee the need for further investigations and genetic studies for the analysis of possible variants and the discovery of further substrates and actions of Corin in order to clarify its effects on cardiovascular homeostasis in physiological and pathological conditions. The knowledge of the effects and mechanisms of action of NPs is fundamental to investigating the electrophysiological consequences, certainly less studied, but which could prove significant considering the increase in electrical conduction and heart rate and, therefore, in oxygen consumption induced by the activation of NPR-A and NPR-B receptors and by the inhibition of phosphodiesterase 3 (PDE3) [43]. Also, in heart valve disease, the diastolic stretching that occurs following blood regurgitation in mitral and aortic insufficiency and the pressure overload typical of aortic stenosis determine the production of BNP, expression of cardiac decompensation and could independently identify individuals more at risk of cardiac events, therefore deserving of closer follow-up and any further cardiological investigations [19, 44]. The increasing importance of NPs in the diagnosis, management, severity, and prognostic implications of HF has recently been the subject of great attention by the major scientific societies concerned with HF—Heart Failure Association of the European Society of Cardiology, Heart Failure Society of America and Japanese Heart Failure Society—in the actions of the so-called Trilateral Cooperation Project [45]. This paper highlights how high levels of NPs, in particular BNP and NT-proBNP, are associated with short- and long-term adverse events with regard to mortality and morbidity, including all-cause and cardiovascular. However, as also specified in our manuscript, there are still no standardized assessments of NPs for HF management. The increasingly frequent use of angiotensin receptor-neprilysin inhibitor (ARNI), whose action is closely linked to that of NPs and the improvement of cardiac performance following their use, has certainly given new impetus to the interest in these molecules, opening new scenarios of molecular and clinical research.

**Table 1.** **Modifier factors of NPs concentrations**.

Factors that decrease serum levels of NPs	Factors that increase serum levels of NPs
Obesity	Advanced age
Acute pulmonary edema	Kidney disease
Constrictive pericarditis	Acute coronary syndrome
Cardiac tamponade	Right ventricular dysfunction
Black individuals	Pulmonary hypertension
Genetic polymorphisms (polymorphisms in the NPPB gene)	Pulmonary embolism
Increased androgencitiy in women	Neprilysin inhibitor therapy (transient increase of BNP levels when treatment is started) *
Hypercortisolism	Cardiotoxic drugs
Insulin resistance	Atrial fibrillation (and other arrhythmias)
Certain medications	Hyperdynamic conditions (sepsis, hyperthyroidism, anemia)
Neprilysin inhibitor therapy (NT-proBNP levels tend to decrease with treatment) *	Valvular diseases
	Genetic polymorphisms (Genotype GG rs198389 and NPPA polymorphisms rs5068 or rs198358)

NPs, natriuretic peptides; NT-proBNP, N-terminal prohormone of BNP; BNP, brain natriuretic peptide. *See Section 4.1 for further discussion. NPPB, natriuretic Peptide B; NPPA, natriuretic Peptide A; GG, Guanina-Guanina.

### 4.2 Troponins

Cardiac troponin, together with NPs, represents one of the most used biomarkers in the follow-up and study of patients with HF. The troponin I (TnI) and troponin T (TnT) are specific blood biomarkers of the heart and, together with troponin C (TnC), constitute the cardiac troponin complex. TnT binds the troponin complex to tropomyosin, facilitating its interaction with actin. TnI, on the other hand, inhibits the interaction between actin and myosin in the absence of calcium ions. In 2018 Moliner *et al*. [46] documented that although there were no substantial differences in high-sensitivity cardiac troponin T (hs-cTnT) values between HFmrEF, HFpEF, and HFrEF heart for prognosis, considering how primary end-points composites cardiovascular death, all-cause death, or HF-related hospitalization, in patients with HFmrEF the risk was significantly higher. In patients with HFrEF, the increase in hs-cTnT reflects the severity, stability, and clinical prognosis proportionally to the magnitude of its increase [47]. In fact, gradual increases in hs-cTnT in patients with chronic HF have been associated with a progressive increase in the incidence of cardiovascular events [48]. Indeed, even in the general population, in the absence of cardiovascular manifestations, alterations in serum cTn levels, albeit minimal, are predictive of HF, coronary events, and cardiac death, as already demonstrated in 2006 by Zethelius *et al*. [49] in 70-years old. In the same period, the Dallas Heart Study documented among the patients enrolled in whom not only HF but also had been excluded LV hypertrophy, diabetes mellitus, and chronic kidney disease, a prevalence of cTnT elevation was 0.7% [50]. Considering these premises or the increase of cardiac troponins (TnT and TnI) not only in HF but also in the preclinical stage, they can represent valid biochemical support in the stratification of the risk of patients with HF. This claim is particularly supported in acute HF. In fact, an increased risk of hospitalization or death was documented in patients with a hs-TnI >23 ng/L, a risk that further increased in hospitalized patients in whom hs-TnI increased during hospitalization compared to patients in whom it remained stable or decreased [51, 52]. Moreover, our group has also documented a reduction in the serum levels of hs-TnT but also of other biomarkers (inflammatory markers interleukin 6 (IL-6), fibrosis markers soluble suppression of tumorigenicity 2 (sST2), NT-proBNP and galectin-3) after depletive therapy and in particular in patients randomized to intravenous furosemide plus hypertonic saline solution [53]. It is, therefore, essential to understand the mechanisms, still little known, that lead to the increase of troponin in HF. The damage suffered by myocardiocytes seems to be independent of ischemic insult and, rather, attributable to multiple factors, as already documented by *in vitro* studies [54]. An increased ventricular preload, the reduction of subendocardial perfusion resulting from LV end-diastolic pressure, and increased myocardial wall stress, norepinephrine, angiotensin II, tumor necrosis factor-α and stretch due to myocardial overload represent conditions capable of damaging the integrity of myocardiocytes and inducing necrosis and apoptosis [48, 55]. The understanding of these mechanisms, therefore, is essential to clarify the role of cardiac troponin complex in HF. It is also reasonable to think that the combined evaluation of hs-cTnT and NT-pro BNP but also of other biomarkers can better identify the patients most at risk.

## 5. Biomarkers of Fibrosis and Inflammation

sST2, galectin-3, and growth differentiation factor-15 (GDF-15) are markers associated with inflammation and fibrosis. These pathological processes are part of the natural history of HF, thus making the dosage of these markers useful in the various stages of the evolution of the pathology itself. Since these markers can be expressed in various tissues, they are considered non-cardiac-specific and, therefore, scarcely usable for diagnostic purposes [56, 57]; however, there is strong evidence that the plasma concentration of these three analytes can provide useful information for prognostic purposes in patients with HF [58, 59].

### 5.1 Suppression of Tumorigenicity 2 (ST2)

Tumorigenesis suppression-2 ligand (ST2L) belongs to the Toll-like receptor group that binds interleukin 33 (IL-33). The IL-33/ST2L complex is a proprietary signaling mechanism of the immune system, also having cardioprotective activities. sST2 is a soluble truncated form of ST2L that is secreted into the circulation and acts as a decoy for IL-33 by inhibiting its positive cardiac effects [60]. sST2 is primarily produced in the lungs by type 2 pneumocytes in response to, among others, fluid overload and pulmonary congestion [61]. Numerous studies have demonstrated the good prognostic value of sST2 in both acute and chronic HF. A meta-analysis of 10 studies including 4835 patients with acute HF found that sST2 levels at admission and discharge were predictive of all-cause death and cardiovascular death and that discharge sST2 predicted rehospitalization for HF [62]. A prospective cohort study by Wang *et al*. [63] enrolled 331 patients with acute HF that were divided into 3 subgroups according to sST2 levels. The patients were followed up for a median period of 21 months for the development of the primary endpoint (cardiovascular death). During the follow-up period, 63 participants died. The study demonstrated how patients with higher sST2 levels had lower left ventricular ejection fraction, higher New York Heart Association (NYHA) classification, and NT-proBNP levels. Multivariate analysis also revealed that sST2 and NT-proBNP were independent risk factors for the primary outcome in all patients with acute HF [63].

Another study evaluated the prognostic value given by serial measurements of sST2 in patients with acute HF. van Vark* et al*. [64] enrolled 496 patients with acute HF by repeatedly measuring plasma sST2 levels over a 1-year follow-up. The primary endpoint was the composite of all-cause mortality and HF rehospitalization. The median baseline ST2 level was 71 ng/mL. During the median follow-up of 325 days, 188 patients reached the primary endpoint of all-cause death or readmission for HF. This corresponds with an incidence rate of 55.9% patient-years for the primary endpoint. In the highest quartile of baseline ST2, 50 patients reached the primary endpoint compared with 22 patients in the lowest quartile of ST2. All-cause mortality was also higher in the highest ST2 quartile compared with the lowest ST2 quartile. This was similar for cardiovascular mortality. This study demonstrates that baseline ST2 levels, especially repeated ST2 measurements, are a strong and independent predictor of the composite endpoint of all-cause mortality or readmission for HF during 1-year follow-up in patients admitted with acute HF [64]. Song *et al*. [65] evaluated the prognostic value of sST2 in patients with chronic HF with reduced (HFrEF), mean (HFmrEF), and preserved (HFpEF) ejection fraction, concluding that higher levels of sST2 measured at ward admission correlated with an increased risk of death from all causes and rehospitalization for HF in patients with HF regardless of ejection fraction. Predicting the efficacy of sST2 on outcomes was higher for HFpEF as compared to HFrEF, but the association between sST2 and outcomes in HFmrEF was not statistical [65]. The prognostic value of sST2 was also confirmed in chronic HF, as demonstrated by a meta-analysis conducted on 5051 patients, which concluded that sST2 is a predictor of both all-cause death and cardiovascular death in chronic HF outpatients [66]. A recent study attempted to establish the prognostic cut-off values ​​of sST2 plasma concentrations in chronic HF, differentiating them by sex. The study concluded that the optimal prognostic cut-off was lower in women than in men (28 vs. 31 ng/mL) [67]. Measurement of ST2 levels as well as other markers of inflammation had been included in the 2017 (American College of Cardiology) ACC/ American Heart Association (AHA) guidelines for HF management as additional risk stratification markers with a class II level of evidence B indication [68]. In the latest edition of the same guidelines, dated 2022, however, this indication was removed. Further studies are probably needed in order to improve the reliability of this marker, particularly by removing the possible confounding factor of extracardiac production of the marker itself.

Lupón *et al*. [69], in a study in which 876 patients with chronic HF were recruited (The Barcelona Study), studied different combinations of biomarkers, including NT-proBNP, ST2, and high-sensitivity troponin T (hsTNT) to determine the relative role of each in risk stratification of chronic HF. All 3 biomarkers were incorporated into a model with 11 established risk factors: age, sex, ischemic etiology, left ventricular ejection fraction (LVEF), NYHA functional class, diabetes mellitus, estimated glomerular filtration rate, sodium level, hemoglobin, treatment with beta-blockers and angiotensin-converting enzyme inhibitors/angiotensin receptor blockers. The different analyses in this study produced 3 relevant results. First, NT-proBNP added to hsTnT and ST2 did not improve prognostic accuracy or reclassification indices. Second, NTproBNP increased prognostic discrimination only in patients with hsTnT or ST2 levels below the cut-off point. Third, the combination of hsTnT and ST2 identified more deaths during follow-up than the combination of the 3 biomarkers. Taken together, these main findings suggest that the pathways identified by ST2 and hsTnT profoundly influence mortality in the context of chronic HF, whereas information in combination with NPs may be redundant [70]. The investigators used these data to develop a new HF risk calculator, the Barcelona Bio-HF calculator [71]. This calculator includes 3 complementary commercially available biomarkers that provide information on myocyte necrosis (hs-TnT); fibrosis, remodeling, and inflammation (ST2); and inflammation (ST2) and chamber deformation (NT-proBNP). The calculator was developed with 8 models that include none, 1, 2, or 3 of the biomarkers, allowing it to be used with any combination of biomarkers and allows to quickly and easily calculate the 1-, 2-, and 3-year prognosis as well as life expectancy.

### 5.2 Galectin-3

Galectin-3 is a versatile protein orchestrating several physiological and pathophysiological processes in the human body. Galectin-3 is differentially expressed depending on the tissue type; however, its expression can be induced under conditions of tissue injury or stress. Galectin-3 overexpression and secretion are associated with several diseases and are extensively studied in the context of fibrosis, HF, atherosclerosis, and diabetes mellitus [72]. In the PRIDE study, patients with HF had higher levels of galectin-3 compared with those without, but for the diagnosis of HF, NT-proBNP outperformed galectin-3 [73]. Galectin-3 may have a prognostic role in predicting long-term mortality in patients with acute HF, as described by Lala* et al*. [74], where patients with high baseline galectin-3 values ​​(>16.5 ng/mL) had a significantly worse survival profile over the 18-month follow-up for all-cause mortality. Its prognostic importance has also been described in patients with chronic HF, where it was considered as a predictor of ventricular remodeling and long-term mortality in the study conducted by Lok* et al*. [75]. The study enrolled a total of 240 patients with NYHA class III and IV HF to whom NT-proBNP and Galectin-3 levels were measured and were subsequently divided into 3 subgroups taking into account the progressive change in left ventricular end-diastolic volume (LVEDV). Patients in whom LVEDV was demonstrated to decrease over the course of the study had significantly lower levels of galectin-3 than patients in whom LVEDV remained stable or increased over time, while no significant changes were observed in NT-proBNP levels. In addition, galectin-3 was a significant predictor of mortality after long-term follow-up. This data was also confirmed by a recent meta-analysis by Wu* et al*. [76], which included 19 studies with a total of 9217 patients in which galectin-3 proved to be a significant marker of mortality in all patients with HF and especially in the acute HF group.

### 5.3 Growth Differentiation Factor 15 (GDF-15)

GDF-15 belongs to the group of transforming growth factor-beta and is partially expressed in numerous organs under disease-free conditions. Under pathological conditions, GDF-15 is most highly expressed in response to ischemic, mechanical, oxidative, or inflammatory stresses. Because of these characteristics, GDF-15 has been considered and analyzed as a biomarker of prognosis in various diseases, including HF, myocardial infarction, atrial fibrillation, diabetes mellitus, and various cancers [77]. The role of GDF-15 in HF has been explored in numerous studies that have focused on defining its prognostic ability against other biomarkers. A study by Gürgöze *et al*. [78] found that elevated GDF-15 values mostly correlated with an increased overall risk of all-cause mortality and rehospitalization for acute HF, independent of other biomarkers, especially NT-proBNP. In another study, GDF-15 was considered in patients who were admitted to the hospital with a diagnosis of acute HFpEF. In this group of patients, GDF-15 levels measured within 48 hours of ward entry were a strong prognostic factor for the risk of rehospitalization for HF at one-year, being higher even than NTproBNP [79].

### 5.4 Matrix Metalloproteinases and Tissue Inhibitors of Metalloproteinases

Matrix metalloproteinases (MMPs) are proteolytic enzymes that participate in the processing of extracellular proteins in the myocardium. The extracellular matrix is ​​then regulated by the continuous activity of MMPs counter-regulated by their tissue inhibitors (TIMPs). The lack of balance between the levels of MMPs and TIMPs results in a constant state of activation of MMPs within myocardial tissue contributing to the process of remodeling of the cardiac chambers as part of the development of chronic HF [80]. During the progression from compensated hypertrophy to HF, it was demonstrated that the levels of MMPs were progressively increased while there was inadequate control by TIMPs; specifically, it was described as MMP-1, -2, -3, -9, -13, and -14 were upregulated while tissue inhibitors of metalloproteinase-1 and -2 were enhanced and TIMP-4 was decreased in comparison to control [81]. Circulating levels of TIMP2 have also been correlated with systolic function in patients with hypertrophic cardiomyopathy, as it was elevated in patients with systolic dysfunction but not in those with preserved systolic function, whereas TIMP-1 levels were elevated in both groups [82]. Among all MMPs, it was seen that higher levels of MMP-2 are correlated with patients with a worse prognosis for HF (NYHA class II–IV) [83], as well as an increased risk of death or hospitalizations for HF [84]. In a recent study, it has also been proposed as a future pharmacological target in patients with HF [85].

## 6. Markers of Renal Damage

Renal dysfunction is frequently present in patients with HF and is associated with a worse prognosis. This is true for both patients with HFrEF and HFpEF [86]. The condition in which renal failure sets in as part of HF has been termed “cardiorenal syndrome”. The main driving force of renal failure in HF is probably hemodynamic derangement, with both reduced renal perfusion and increased venous pressure as the most important driving forces. In addition, renal failure consists not only of reduced renal flow and, thus, reduced filtration capacity but also involves increased pressure at the glomerular level and tubular hypoxic damage, resulting in loss of glomerular integrity and tubular necrosis [87]. Therefore, since a strong connection between the cardiovascular and renal systems has been established, it was hypothesized that some markers of renal damage could be used for prognostic purposes also in patients suffering from HF. In addition to the main markers of renal damage, such as blood urea nitrogen, creatinine, and glomerular filtration, which are often altered in patients with HF and which are frequently monitored in patients taking diuretics, recent studies have explored some new markers of renal damage in order to establish their possible prognostic value in patients with HF.

### 6.1 Cystatin-C

Cystatin-C (Cys-C) is a cysteine proteinase inhibitor produced by nucleated cells, freely filtered by the glomerulus, and then reabsorbed by the proximal tubules, where it is catabolized. Some studies dating back to 2005 have already described that high serum levels of Cys-C were directly associated with an increase in mortality from cardiovascular causes [88] and that cystatin-C concentration is an independent risk factor for HF in older adults and appears to provide a better measure of risk assessment than the serum creatinine concentration [89]. In a more recent meta-analysis, it was confirmed that elevated Cys-C levels are possibly associated with an increased risk of all-cause mortality and rehospitalization in HF patients and that this increase is probably independent of creatinine or estimated glomerular filtration rate (eGFR) [90]. In a study that compared the prognostic values ​​of Cys-C and NT-proBNP, it was described that in patients with acute HF and normal or slightly reduced renal function, the prognostic performance of Cys-C could be superior to other classical markers, including NT-pro-BNP [91]. Cys-C has been shown to maintain its prognostic value of poor outcomes even in patients admitted with HF with preserved ejection fraction despite normal or mildly reduced renal function [92].

### 6.2 Neutrophil Gelatinase-Associated Lipocalin (NGAL)

Neutrophil gelatinase-associated lipocalin (NGAL) Kidney is a protein belonging to the lipocalin family initially isolated in activated neutrophils with iron binding capacity and bacteriostatic activity. Subsequent studies have isolated NGAL in numerous tissues and specifically at the level of renal tubular cells with high production in case of inflammatory and/or ischemic stimuli [93]. Recent studies have investigated the role of NGAL as an additional marker of acute renal failure attributing to it an important role as a marker of early renal damage [94]. Subsequent studies demonstrated that urinary NGAL levels were significantly increased in patients with HF and chronic kidney disease and that this increase was positively associated not only with other markers of renal damage such as reduced glomerular filtration rate (GFR) and increased urinary albumin excretion (UAE) but also an increase in serum NT-proBNP values [95]. In a study by van Deursen* et al*. [96] that enrolled 562 patients with HF, higher plasma NGAL levels were independently associated with an increased risk of all-cause mortality in patients with and without chronic kidney disease. The same study showed that NGAL is a stronger predictor for mortality than the established renal function markers eGFR and cystatin-C [96].

### 6.3 Kidney Injury Molecule-1 (KIM-1) E N-Acetyl-ß-d-Glucosaminidase (NAG)

Kidney injury molecule-1 (KIM-1) and N-acetyl-ß-d-glucosaminidase (NAG) are two markers of renal damage expressed at the proximal tubule level that have recently been studied in HF patients. A study that considered patients with chronic HF urinary analysis showed that KIM-1 was significantly elevated in HF patients compared with healthy controls; furthermore, KIM-1 increased significantly with worsening of left ventricular function and severity of NYHA class. NAG instead showed a weaker response but correlated significantly with left ventricular dysfunction and more severe clinical condition. Also, both were predictors of all-cause mortality and the composite of all-cause mortality and rehospitalization for HF [97]. In 2130 patients participating in the GISSI-HF trial (Gruppo Italiano per lo Studio della Sopravvivenza nell’infarto Miocardico Heart Failure trial), increased tubular markers NGAL, KIM-1 and NAG were related to a poorer outcome (combined endpoint of death and HF hospitalization) even with a normal renal function [98].

## 7. Adrenomedullin

Adrenomedullin (ADM) is a hormone primitively isolated from cells of the medullary portion of the adrenal gland, having natriuretic and vasodilatory activity; however, its expression is ubiquitous in various organs and tissues, including the cardiovascular, renal, pulmonary, cerebrovascular, gastrointestinal, and endocrine systems. ADM also has antihypertrophic, anti-apoptotic, antifibrotic, antioxidant, and angiogenesis effects. Given its poor *in vitro* stability due to its short half-life, it is preferable to dose a fragment of its precursor, the mid-regional pro-ADM (MR-proADM), which corresponds to the plasma concentration of ADM. A study dating back to 1995 described how ADM levels were increased in patients with HF, possibly due to volume overload and activation of the sympathetic nervous system [99]. In the Biomarkers in Acute Heart Failure (BACH) trial, a multicenter study that enrolled 1641 patients presenting to the emergency area with dyspnea, MR-proADM was shown to be a better marker of 90-day mortality risk than BNP [100]. In a second study derived from the BACH trial, which took into consideration patients diagnosed with acute HF in the emergency area, MR-proADM, evaluated individually or in combination with copeptin, was confirmed as the best 14-day mortality marker compared to NPs and troponin [101]. A further study by Gegenhuber *et al*. [102] compared the prognostic ability of some markers, including MR-proADM versus BNP in 137 patients with acute HF, finding that the predictive ability of MR-proADM was superior to BNP in assessing the risk of death from all causes to one-year. MR-proADM has therefore demonstrated a good ability to select a class of patients with acute HF at high risk of mortality compared to the other biomarkers but has not yet found application in the clinical setting.

## 8. MicroRNA

Micro RNAs (miRNAs) are small RNA molecules of about 22 nucleotides that act primarily as regulators of gene expression at both the transcriptional and post-transcriptional levels; they have the ability to bind to messenger RNA (mRNA) molecules and lead to their inhibition or to the degradation of the mRNA itself [103], thus having a fundamental role in numerous biochemical processes. Their discovery dates back to 1993 when the first, *lin-4*, was identified in the nematode *Caenorhabditis elegans * [104]. Since then, many miRNA molecules have been isolated in various tissues and biological fluids, and due to their varied expression and ability to interact with numerous physiological and pathological processes, they have been the subject of numerous research studies. At the cardiac level, miRNAs play a fundamental role since the embryonic stage by promoting the differentiation of stem cells into specific cardiac muscle cells, in parallel they are important in the differentiation into cells that constitute the specific conduction tissue, thus participating in the regulation of the cardiac action potential [105]. Therefore, given this variety of expression, miRNAs have been taken into consideration in the most common cardiac pathologies, such as acute myocardial infarction, atrial fibrillation, and HF, in order to establish their possible role in the pathogenesis or evolution of these disorders. In HF patients, miRNA 21 (miR-21), miR-1, miR-23a, miR-142-5p, miR-126, miR-29, miR-195, and miR-499 were found to be the most frequent miRNAs associated with conditions such as hypertrophy heart and fibrosis leading to the development of the pathology itself [106]. In a recent meta-analysis, miRNAs demonstrated good sensitivity and specificity in identifying patients with chronic HF, albeit not superior to conventional markers (NT-proBNP and BNP). In the same study, it was then analyzed which of these miRNAs could have the ability to select patients with HFpEF compared to patients with HFrEF. In the group of patients with HFrEF, eight miRNAs (miR-18b, miR-129, miR-423, miR-320a, miR-22, miR-92b-, miR-675, and miR-21) were identified that were most highly expressed at the serum and plasma levels, on the other hand, seven miRNAs (miR-424, miR-206, miR-328, miR-30c, miR-221, miR-375, and miR-19b) were identified for the HFpEF patient group, most of which were downregulated compared to healthy controls [107]. In a small study by Tijsen *et al*. [108], it has been described that circulating levels of miR423-5p are increased only in patients with clinical HF and that miR423-5p levels are related to NT-proBNP and NYHA classification. Another study conducted by Goren *et al*. [109] measured the serum levels of 186 microRNAs in the sera of 30 stable chronic systolic HF patients and 30 controls, demonstrating how, among these, only 4 were expressed to a greater extent in the group of patients with HF (miR-423-5p, miR-320a, miR-22, and miR-92b) with a specificity and sensitivity of 90%, and there was a correlation with important prognostic parameters including elevated serum BNP levels, a wide QRS, and dilatation of the left ventricle and left atrium. Zhang *et al*. [110] conducted a study focusing on defining the diagnostic and prognostic capabilities of miRNA-21, demonstrating that it was significantly increased in the group of patients affected by HF and furthermore, during the follow-up, it proved to be a valid prognostic marker in predicting the death from all causes and the rehospitalization rate. From the listed studies, it is clear that the current and, above all, future possibilities of using miRNAs in the clinic are many and of great interest as they can be able to define clusters of patients with certain clinical characteristics as well as being a valid aid in the diagnosis and in the prognosis of patients with HF together with classical markers. MiRNAs also represent a possible future therapeutic target with the possibility of altering the physiological or pathological processes in which they play a central role, thus inhibiting their expression or, on the contrary, synthesizing molecules that can camouflage their role [111]. However, there are currently numerous limitations to the use of miRNAs, such as their overlap in various cardiac pathologies, the difficulties of isolation and sampling, the lack of normalized parameters, and the high costs which currently make their routine use difficult.

## 9. Conclusions

**Fig. 1.**
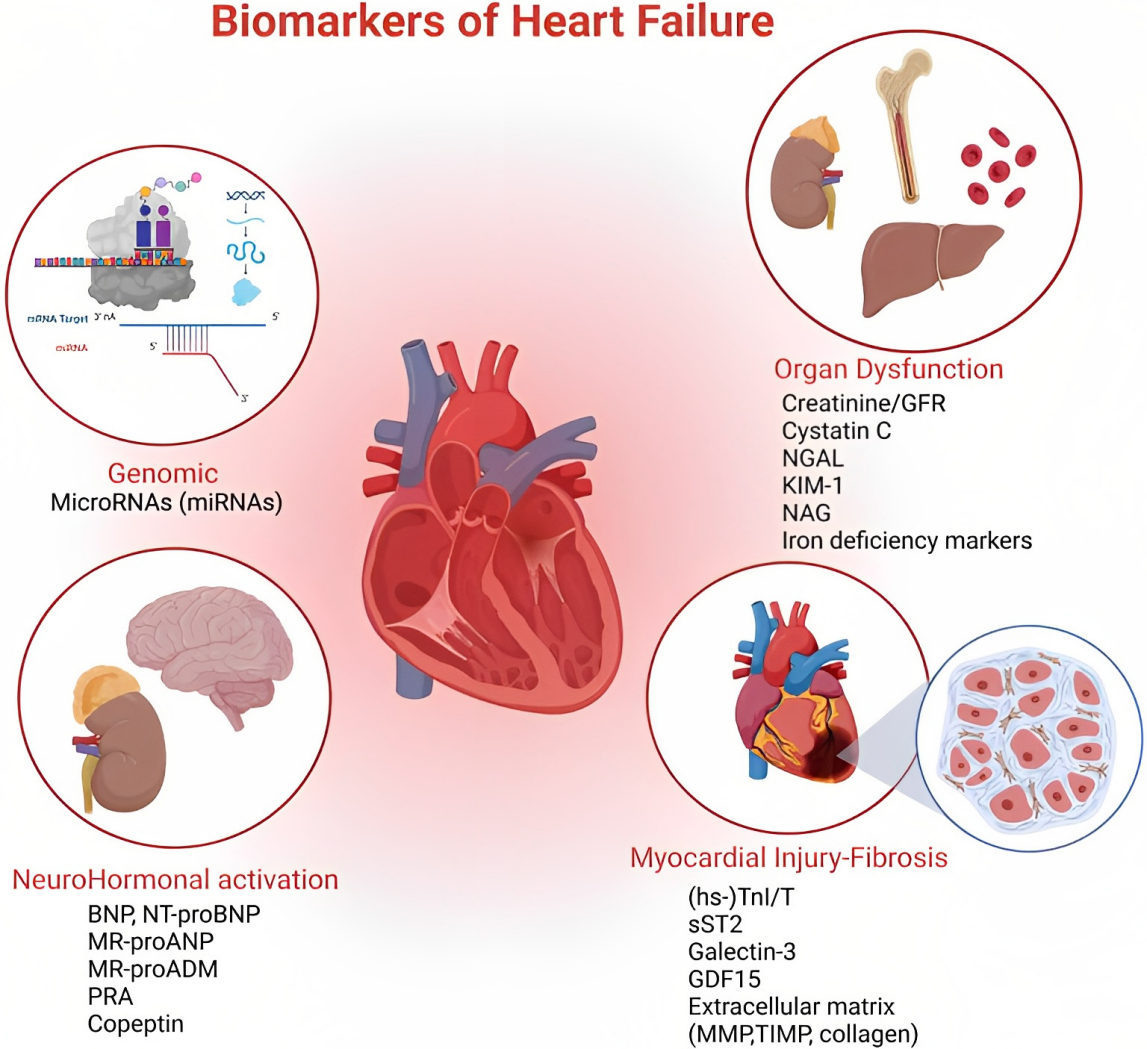
**At the top of the figure, on the left, we show how miRNAs act as regulators of gene expression at both transcriptional and post-transcriptional levels**. Top right we show the effects on different target organs of HF, also emphasizing the role of iron deficiency. This part of the figure, together with the lower left one wants to emphasize the syndromic character of the disease and the pathophysiological implications. Finally, at the bottom right, a summary of direct and indirect myocardial damage, remaining the heart at the center of the complexity of the clinical picture that inevitably involves all tissues. HF, heart failure. **Organ Dysfunction**: Creatinine/GFR, glomerular filtration rate; NGAL, neutrophil gelatinase-associated lipocalin; KIM 1, kidney injury molecule 1; NAG, N-acetyl-ß-d-glucosaminidase. **NeuroHormonal Activation**: BNP, brain natriuretic peptide; NT-proBNP, N-terminal prohormone of brain natriuretic peptide; MR-proANP, mid-regional-fragment of pro-atrial-natriuretic-peptide; MR-proADM, mid-regional Pro-adrenomedullin; PRA, plasma renin activity. **Miocardial Injury-Fibrosis**: hs-TnI/T, high sensitivity Troponin I (TnI), Troponin T (TnT); sST2, soluble suppression of tumorigenesis-2; GDF15, growth differentiation factor 15; MMP, matrix metalloproteinases; TIMP, tissue inhibitor of metalloproteinase.

No biomarkers (Fig. 1) at present appear so sensitive and specific as to represent the ideal molecule for follow-up of patients most at risk of developing adverse events. However, the combined assessment of multiple biomarkers together with other parameters (electrocardiographic, echocardiographic, demographic) might, with further research, be shown to provide valuable support for the interpretation of the higher or lower clinical risk of HF patients and the need for more accurate follow-up and possible earlier therapeutic intervention. The line of research concerning the role of miRNAs as biomarkers of HF, in which our group is also currently engaged, has recently taken on great scientific importance because of their great variability in expression and the possibility of being able to divide patients into specific subgroups, thus improving both clinical and therapeutic approaches, and may thus represent a possible innovative and reliable marker of the future. We anticipate the requirement for more investigations to uncover additional biomarkers and substrates will be needed to elucidate the impact on cardiovascular homeostasis under physiological and pathological conditions. Finally, we anticipate that the knowledge gained regarding the biology of biomarkers will be translated into diagnostic and/or therapeutic agents in the future for the benefit of HF patients.
